# Immature granulocytes can help the diagnosis of pulmonary bacterial infections in patients with severe COVID-19 pneumonia

**DOI:** 10.1186/s40560-021-00575-3

**Published:** 2021-09-20

**Authors:** Thomas Daix, Robin Jeannet, Ana Catalina Hernandez Padilla, Philippe Vignon, Jean Feuillard, Bruno François

**Affiliations:** 1 Inserm CIC 1435, Dupuytren Teaching Hospital, 87000 Limoges, France; 2 UMR 1092, Faculty of Medicine, University of Limoges, 87000 Limoges, France; 3 Réanimation Polyvalente, CHU Dupuytren, 2 Avenue Martin-Luther King, 87042 Limoges, France; 4 UMR CNRS 7276, INSERM 1262, Faculty of Medicine, University of Limoges, 87000 Limoges, France; 5 Hematology Laboratory, Dupuytren Teaching Hospital, 87000 Limoges, France

**Keywords:** Biomarker, COVID-19, Immature granulocytes, Secondary infections, Ventilator-associated pneumonia

## Abstract

During COVID-19, immature granulocyte (IG) concentration is heterogeneous with higher concentrations than those found in bacterial sepsis. We investigated the relationship between IG levels at ICU admission and on days 7 (± 2) and 15 (± 2) and associated pulmonary bacterial infections in intensive care unit (ICU) patients hospitalized for an acute respiratory distress syndrome (ARDS) related to SARS-CoV-2. Patients with associated pulmonary bacterial infection had a peak of IGs. IG thresholds of 18% or 2 G/L allowed discriminating patients with ventilator associated pneumonia with 100% sensitivity and specificity. Our study supports that IGs could help identifying pulmonary bacterial infections in this population.

**Supplementary Information:**

The online version contains supplementary material available at 10.1186/s40560-021-00575-3.

## Introduction

Patients hospitalized in the intensive care unit (ICU) with an acute respiratory distress syndrome (ARDS) related to SARS-CoV-2 frequently develop associated pulmonary bacterial infections, including ventilator-associated pneumonia (VAP) which diagnosis is challenging in this clinical setting [1, 2]. In bacterial sepsis and severe COVID-19, the myeloid cell compartment is dysregulated and circulating levels of immature granulocytes (IG) may increase [3, 4]. The range of IG increase appears highly variable in COVID-19 [4, 5]. We previously showed that septic patients exhibit higher IG levels than patients with severe SARS-CoV-2 infection [3, 5]. We hypothesized that IG levels heterogeneity could be related to the development of pulmonary bacterial infections in patients mechanically ventilated for a SARS-CoV2-induced ARDS.

## Methods

**Fig. 1:**
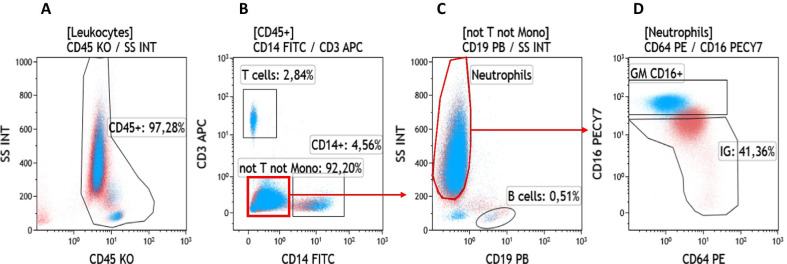
Example of flow cytometry biparametric histograms showing the gating strategy used to identify immature granulocytes (IG) in peripheral blood of COVID-19 patients. The examples shown here are merged data of one same patient on day 0 (in blue dot) and day 7 (in red dot) to illustrate gating strategy. **A** Hematopoietic cells were selected on specific morphological parameter (Side Scatter channel, reflecting the granularity of the cytoplasm) and expression of CD45 (a pan-leukocyte marker). **B** Hematopoietic cells positive for CD14 (monocyte maker) were considered as monocytes, and the ones positive for CD3 (T cell marker) as T lymphocytes. The red square corresponds to cells that are negative for these two markers (Not T not mono). **C** Side scatter (cytoplasm granularity) and CD19 (B cell marker) were used to separate the neutrophils (red gate) and the B lymphocytes, respectively. **D** Neutrophils were divided into two subtypes (i) mature granulocytes strongly positive for CD16 (CD16+) and (ii) Immature granulocyte (IG) low or negative for CD16. CD64 was used as an activation marker

Between December 2020 and March 2021, consecutive patients without known immunosuppression who required invasive mechanical ventilation for severe COVID-19 pneumonia were prospectively enrolled. The evolution of peripheral blood leukocyte populations were studied, from ICU admission to day 7 (± 2) and day 15 (± 2). Using flow cytometry, leukocyte populations were discriminated with CD3 for the T cells, CD19 for the B cells, CD14 for the monocytes, and CD16 for the granulocytes. CD45 was used to identify the hematopoietic cells and CD64 was used as a marker of neutrophils activation. Immature granulocytes or “band cells” were characterized as CD45^+^CD3^−^CD19^−^CD14^−^CD16^dim/neg^ (Fig. 1). Monocyte expression of HLA-DR, CD4^+^ and CD8^+^ T lymphocytes counts were also analyzed.

An independent committee blindly adjudicated the diagnosis of pulmonary bacterial infections during the ICU stay based on clinical findings (fever, new onset of purulent endotracheal sputum or modification of sputum characteristics, auscultation abnormalities, increasing need of oxygen therapy), biological abnormalities (hyperleukocytosis, decreased PaO_2_/FIO_2_), radiological data (new onset or worsening of pulmonary infiltrate), and microbiological documentation. Pulmonary bacterial infections diagnosed within the first 2 days of ICU stay were considered as co-infections, while those diagnosed later were reported as VAP.

## Results

**Fig. 2:**
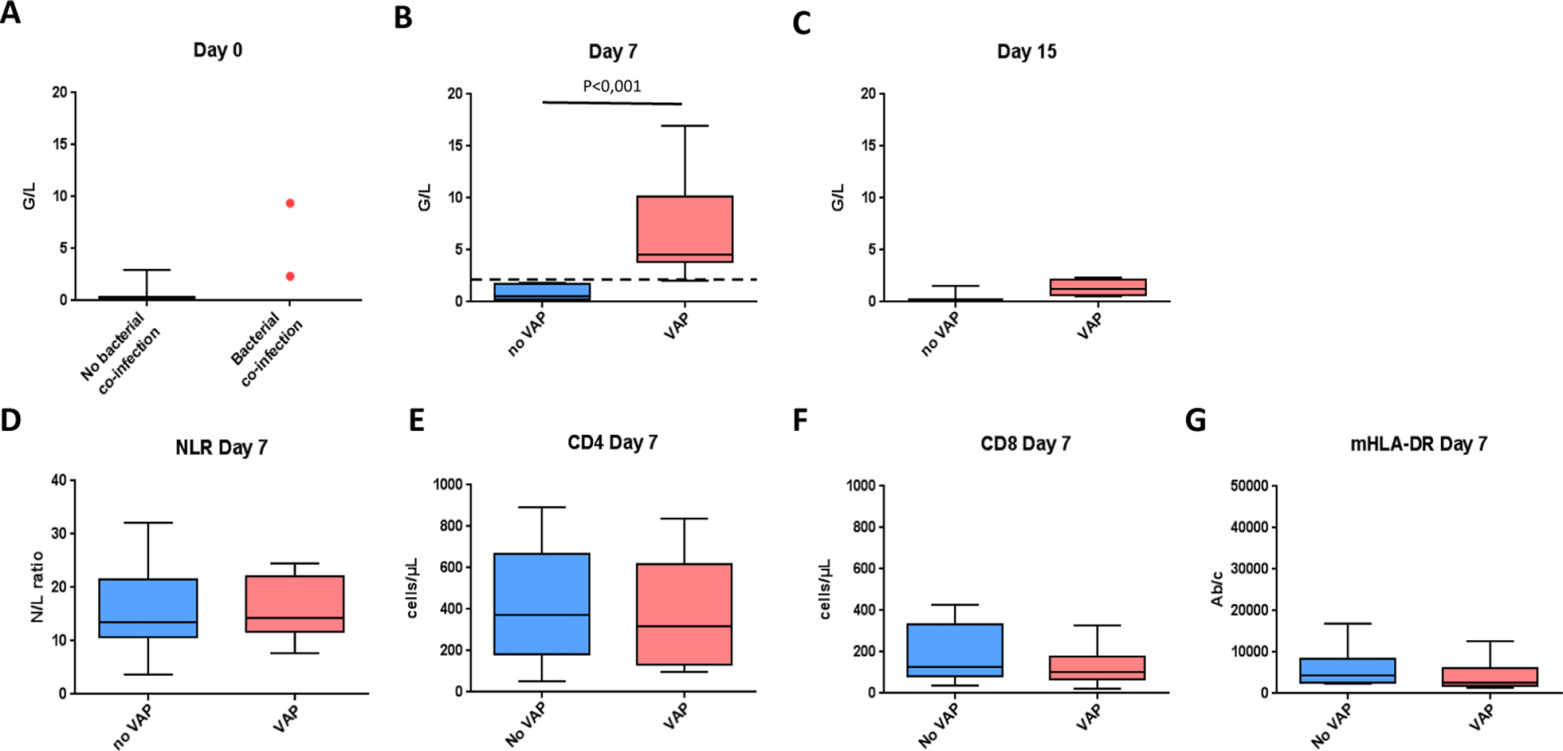
**A** IG number in G/L in peripheral blood at Intensive care unit admission in COVID-19 patients without bacterial co-infection (blue, *N* = 17) vs patients with bacterial co-infection (red, *N* = 2). **B** IG number in G/L in peripheral blood on day 7 (± 2) in patients without VAP (blue, *N* = 7) vs patients with VAP (red, *N* = 10). The dot line represents the threshold separating patients with or without VAP. **C** IG number in G/L in peripheral blood on day 15 (± 2) in patients without VAP (blue, *N* = 3) vs patients that had VAP (red, *N* = 5). **D** Neutrophil to lymphocyte ratio (NLR) on day 7 (± 2) in patients without VAP (blue, *N* = 7) vs patients with VAP (red, *N* = 10). **E** CD4 lymphocyte absolute number in cell/µL on day 7 (± 2) in patients without VAP (blue, *N* = 7) vs patients with VAP (red, *N* = 10). **F** CD8 lymphocyte absolute number in cell/µL at day 7 (± 2) in patients without VAP (blue, *N* = 7) vs patients with VAP (red, *N* = 10). **G** HLA-DR expression on monocytes represented in antibodies bound per cell (Ab/c) on day 7 (± 2) in patients without VAP (blue, *N* = 7) vs patients with VAP (red, *N* = 10). Results from **A** to **G** are represented in boxes (mean and interquartile range) and whiskers (min to max range); circles represent individual values. *p* values were calculated using the Mann–Whitney test comparison

**Fig. 3:**
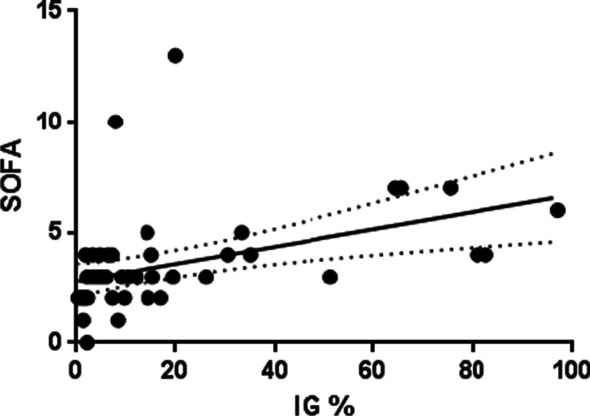
Spearman correlation representation between sequential organ failure assessment (SOFA) score and immature granulocyte frequencies. The plain dark line corresponds to the linear regression and the dash lines correspond to the 95% confidence intervals

Nineteen patients ventilated for severe COVID-19 were studied (72 [63.0–74.5] y.o; SAPS II: 29 [26.0–35.5]; mortality rate: 32%). Severity scores, comorbidities and steroids use were similar, irrespective of the presence of a pulmonary bacterial infection (Table 1). Two patients were admitted to ICU with a pulmonary bacterial co-infection, whereas 10 patients developed VAP (median diagnosis: 6.5 [4.3–7.8] days). On ICU admission, patients without pulmonary co-infection (*n* = 17) exhibited markedly lower circulating IG levels in absolute count (0.40 ± 0.75 G/L) and in percentage (3.22 ± 3.78%) than those with bacterial co-infection (2.30 G/L and 9.37 G/L in absolute count, or 75% and 84% in percentage) (Fig. 2A). In the two patients with bacterial co-infection at admission, IG absolute numbers and frequencies decreased with time (Additional file 1: Figure S1). On day 7, patients with confirmed VAP had a major peak of IG, both in percentage and absolute numbers, when compared to patients without VAP (55.6 ± 26.6% vs 9.0 ± 5.9%, *p* = 0.0001 and 6.9 ± 4.72 G/L vs 0.95 ± 0.75 G/L, *p* = 0.0002; Fig. 2B). IG thresholds of either 18% or 2 G/L allowed discriminating patients with or without VAP with a 100% sensitivity and specificity (Fig. 2B). On day 15, IG levels decreased in patients who developed VAP on day 7, and were close to those observed on ICU admission (8.7 ± 5.6% and 1.75 ± 1.13 G/L; Fig. 2C). IG levels were moderately correlated to the SOFA score (Sequential Organ Failure Assessment) (Fig. 3; Spearman test *r* = 0.62). Total neutrophil count tended to be increased in patients with VAP but without reaching statistical significance. No significant difference was noticed on day 7 in the neutrophil to lymphocyte ratio (NLR) (Fig. 2D). CD4 and CD8 T lymphocyte counts as well as HLA-DR monocyte expression were similar between patients (Fig. 2E, F).

**Table 1:** Study population

	Study population (*n* = 19)	Bacterial pulmonary infection (*n* = 12)	No bacterial infection (*n* = 7)
Demographics			
Age	72 [63;74.5]	72.5 [59.5;73.8]	70 [65;74.5]
Gender			
Male, *n* (%)	13 (68)	10 (83)	3 (43)
BMI, kg/m^2^	26.8 [24.5;31.5]	26.4 [23.4;31.2]	27 [26;29.8]
BMI > 30, *n* (%)	6 (32)	4 (33)	2 (29)
Comorbidities, *n* (%)			
Hypertension	6 (32)	3 (25)	3 (43)
Diabetes	6 (32)	4 (33)	2 (29)
COPD	2 (11)	2 (17)	0
Chronic heart failure	1 (5)	1 (8)	0
Chronic renal failure	2 (11)	1 (8)	1 (14)
Immunosuppression	1 (5)	0	1 (14)
ICU admission			
SAPS II	29 [26;35.5]	29 [26.5;33.5]	33 [26;39]
SOFA score	2 [2;4]	2 [2;4]	3 [2;3]
Days from onset of disease to ICU admission	8 [6;11]	7.5 [4.75;9]	10 [9;15.5]
Steroids before ICU admission, *n* (%)	3 (16)	2 (17)	1 (14)
Mechanical ventilation at ICU admission, *n* (%)	5 (26)	4 (22)	1 (14)
Severe ARDS at ICU admission, *n* (%)	7 (37)	3 (25)	4 (57)
Steroids, *n* (%)	3 (16)	2 (17)	1 (14)
Dead at ICU discharge, *n* (%)	6 (32)	6 (50)	0

*BMI* body mass index, *COPD* chronic obstructive pulmonary disease, *ICU* intensive care unit, *SPAS* Simplified Acute Physiology Score, *SOFA* sequential organ failure assessment, *ARDS* acute respiratory distress syndrome

## Discussion

Our results, even if obtained on a small cohort, suggest a strong association between level of IGs and pulmonary bacterial infection. Provided consolidation, they indicate that the peak of IG observed in case of bacterial associated infection could be a characteristic reaction of COVID-19.

In contrast to previous studies [4, 6, 7], IG levels were not associated with clinical severity in our patients as reflected by the absence of correlation between IG level and SOFA score. Nevertheless, the occurrence of secondary bacterial infection was not documented in these studies [4, 6, 7]. Associated pulmonary bacterial infections affect approximatively 50% of ventilated patients with severe COVID-19 [8]. This incidence appears superior to that observed in influenza or non-viral pneumonia [9]. In the clinical setting of acute viral ARDS, bacterial infection is associated with an increased risk of death [10]. In addition, radiological and clinical criteria are often inconclusive and, the diagnosis as well as the start of antibiotics mainly rely on microbiological documentation [2, 11]. Due to the severity of SARS-CoV-2 pneumonia in ICU patients, the occurrence of an associated bacterial pulmonary infection requires early antibiotic therapy. Nevertheless, inappropriate antibiotic prescription could favor the emergence of multidrug resistant bacteria which could also jeopardize outcome. Therefore, a reliable biomarker would be highly beneficial in the context of the challenging diagnosis of associated bacterial infection in severe COVID-19.

Even if our results need to be validated by further larger scale studies, our proof of concept study supports that IGs could be an interesting biomarker of bacterial over-infections in ICU patients with severe COVID-19, especially since more and more hospitals have access to flow cytometry.

## Supplementary Information

**Additional file 1.** The figure shows the evolution of IG levels in the two COVID-19 patients with bacterial co-infection at admission on day 7 (± 2) and day 15 (± 2).

## Data Availability

Not applicable.

## Abbreviations

ARDS: Acute respiratory distress syndrome
COVID-19: Coronavirus disease 2019
ICU: Intensive care unit
IG: Immature granulocyte
NLR: Neutrophil to lymphocyte ratio
SAPS: Simplified Acute Physiology Score
SOFA: Sequential Organ Failure Assessment
VAP: Ventilator-associated pneumonia
