# Supplementary material for: Immature granulocytes can help the diagnosis of pulmonary bacterial infections in patients with severe COVID-19 pneumonia
Source: J Intensive Care. 2021 Sep 20;9:58. doi: 10.1186/s40560-021-00575-3 (PMC8451732; doi:10.1186/s40560-021-00575-3)
Supplement: Supplementary file 1 — Additional file 1. The figure shows the evolution of IG levels in the two COVID-19 patients with bacterial co-infection at admission on day 7 (± 2) and day 15 (± 2). [file 40560_2021_575_MOESM1_ESM.pdf]

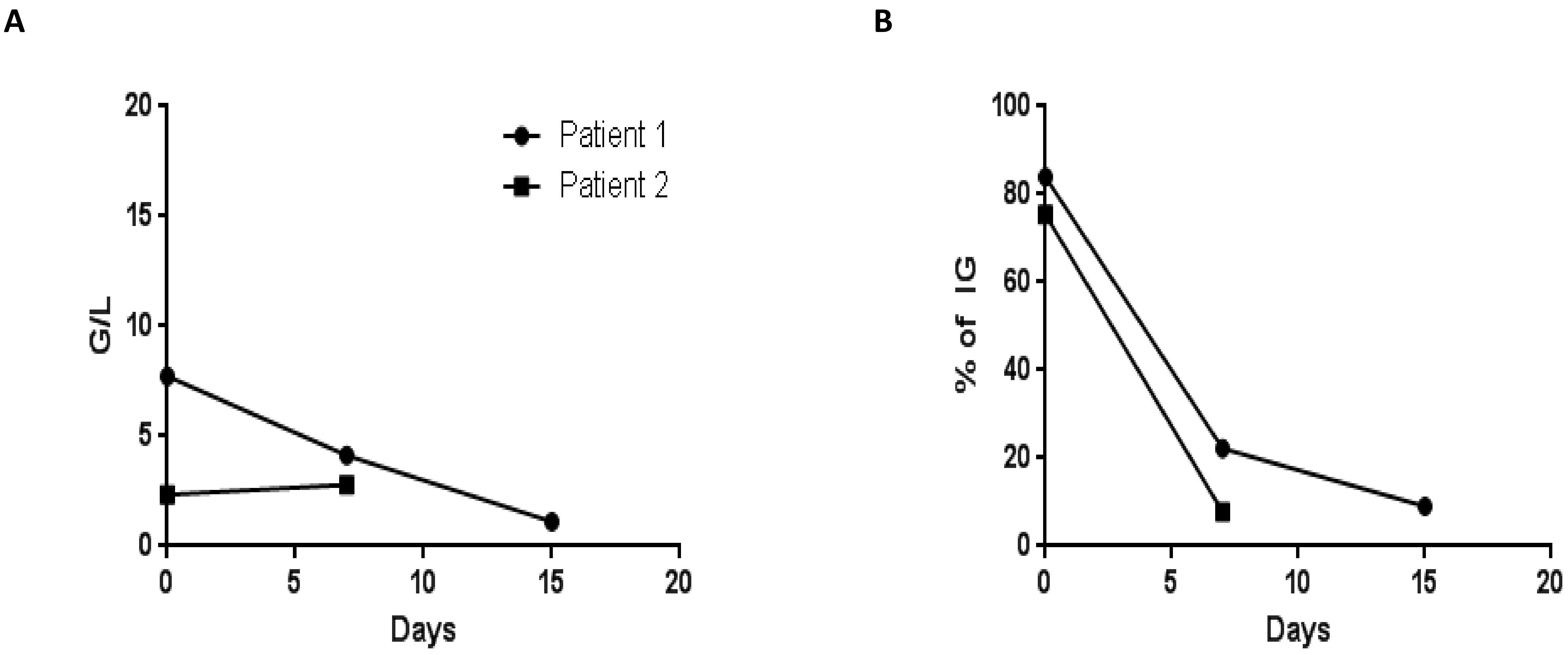

Evolution of IG levels in the two COVID-19 patients with bacterial co-infection at admission on day 7 (±2) and day 15 (±2) when available.
A: IG number in G/L
B: IG frequencies among total leukocytes in percentage.
